# The effects of heterogeneity on stochastic cycles in epidemics

**DOI:** 10.1038/s41598-017-12606-x

**Published:** 2017-10-11

**Authors:** Francisco Herrerías-Azcué, Tobias Galla

**Affiliations:** Theoretical Physics, School of Physics and Astronomy, The University of Manchester, Manchester, M13 9PL United Kingdom

## Abstract

Models of biological processes are often subject to different sources of noise. Developing an understanding of the combined effects of different types of uncertainty is an open challenge. In this paper, we study a variant of the susceptible-infective-recovered model of epidemic spread, which combines both agent-to-agent heterogeneity and intrinsic noise. We focus on epidemic cycles, driven by the stochasticity of infection and recovery events, and study in detail how heterogeneity in susceptibilities and propensities to pass on the disease affects these quasi-cycles. While the system can only be described by a large hierarchical set of equations in the transient regime, we derive a reduced closed set of equations for population-level quantities in the stationary regime. We analytically obtain the spectra of quasi-cycles in the linear-noise approximation. We find that the characteristic frequency of these cycles is typically determined by population averages of susceptibilities and infectivities, but that their amplitude depends on higher-order moments of the heterogeneity. We also investigate the synchronisation properties and phase lag between different groups of susceptible and infected individuals.

## Introduction

It is now widely recognised that noise and uncertainty play an important role in modelling biological systems. Traditional approaches to modelling phenomena in biology^1^ are often based on deterministic ordinary or partial differential equations, and do not aim to describe stochasticity. In order to capture epistemic uncertainty, static or dynamic noise variables are introduced in more modern mathematical biology. This randomness reflects the lack of detailed knowledge about phenomena at finer scales than described by the model at hand; any modelling approach necessarily operates at a set scale (e.g. cell, individual, or population), and does not capture in detail the processes at smaller scales. These are ‘emulated’ through effective randomness. Different types of such noise are frequently found in models of biological phenomena, including intrinsic demographic noise, extrinsic stochasticity, parameter uncertainty or heterogeneity between different types of interacting entities^2,3^. Some of these random variables are static and do not evolve in time, others are described by dynamic time-dependent noise. Intrinsic noise, due to the stochastic dynamics of a system, has lately been the focus of many studies (see for example^4–6^). Extrinsic or parametric noise, due to variations, heterogeneity or uncertainties in the parameters or the environment surrounding the process, has received similar attention (e.g^7–9^). To be able to adequately describe biological systems, however, it may be necessary to account for both these uncertainties which contribute to the noisy dynamics.

In the modelling of epidemics this is of particular importance. The infection process, driven by serendipitous contacts, is inherently stochastic, and heterogeneity in susceptibility to a disease or infectiousness of different individuals are known to exist and play a role in viral spread. Genetic differences that result in heterogeneous susceptibilities to a disease have been suggested to play an important role^10,11^, and variation in viral reproduction from host to host have been observed in ref.^12^. Behavioural, structural or contact differences between individuals are inevitable, but we focus our study on the former type of heterogeneity. However, the better part of the existing work focusing on heterogeneity of this type, does not explicitly seek to capture demographic noise. Instead one often assumes infinite populations and deterministic dynamics. This approach is often taken outside epidemics as well. Much existing work studies *individual* sources of uncertainty, heterogeneity and noise in isolation, but not their interacting together. A notable exception is the modelling of gene regulatory networks, in which the interaction of intrinsic and extrinsic noise is actively studied, see e.g. refs^13–15^.

The effects of intrinsic noise have been recognised in recent years. In models with demographic processes, for example, intrinsic stochasticity has been seen to lead to sustained quasi-cycles^16–19^ in parameter regimes in which a deterministic model would converge to a stable fixed point. These quasi-cycles have been identified not only in models of epidemic spread, but also in other instances of population dynamics, including in genetic circuits, evolutionary systems and in game theory^20–23^. Heterogeneity has been and is being considered in epidemics as well. Age structure is studied for example in refs^24,25^, seasonally changing infection rates in refs^26,27^, variation in infectivity and/or susceptibility are addressed in refs^28–32^, spatial structure has been approached in refs^33–36^, and epidemics on static and dynamic networks are studied in refs^7,37–41^. Heterogeneity has been found to generate outbreaks that propagate hierarchically^38,42^, grow faster than in homogeneous populations^39^, and have a lower total number of infected individuals^43,44^.

Much of this work, whether describing a well-mixed population, a compartmented or structured one, is based on variants of the celebrated susceptible-infective-recovered (SIR) model. They can be described either by deterministic differential equations, or as a stochastic process involving a population of discrete individuals. In the former approach the population is effectively assumed to be infinite, so that the timing of stochastic infection, recovery or birth-death events ‘averages’ out, and smooth laws for the time evolution of the population are obtained. The latter approach explicitly captures the intrinsic randomness of infection, recovery and demographics. The population is taken to be finite, and its state discrete. The model evolves through discrete events (e.g. infections). In the simplest case this defines a Markovian random process, which often can be analysed further mathematically, at least to a good approximation. Starting from the master equation in a well-mixed population a set of stochastic differential equations can be derived in the limit of large, but finite populations^45^. These can then be studied further within the ‘linear-noise approximation’ (LNA)^46^. The mathematics are tractable and the corresponding theory is now well established. While remarkably powerful, this approach so far has mostly been used for well-mixed populations. The linear-noise approximation has also been applied to networked systems with contact heterogeneity (see e.g refs^19,47^), but progress is then much harder and often relies on further moment-closure approximations.

The aim of our work is to introduce agent-to-agent heterogeneity into the SIR dynamics in a finite well-mixed population. This provides a middle ground between homogeneous well-mixed models and an explicitly networked population. At the same time, we maintain tractability and are able to characterise stochastic effects in finite populations via the linear-noise approximation. This allows us to systematically investigate the combination of parameter heterogeneity and demographic noise. We divide the population of agents into *K* different groups of susceptible individuals, where members of different groups have different susceptibilities. Similarly, in our model there are *M* classes of infective individuals, with each class representing a different propensity to pass on the disease. This follows the lines of ref.^32^, but we explicitly focus on the combination of heterogeneity and intrinsic noise. Intrinsic stochasticity had not been included in ref.^32^.

Our paper is organised as follows: In the following section we describe our model in detail. As a baseline we then construct the deterministic rate equations. They describe the deterministic dynamics in the limit of infinite populations, and are required to carry out the LNA. The most natural deterministic description will generally involve *K* + *M* coupled non-linear equations (one for each subclass in the population). We discuss when and how these can be reduced to a smaller set of equations for aggregate quantities. In the next section we perform then the linear-noise approximation and use this approximation to characterise the fluctuations about deterministic fixed points. In particular we set up the theory to obtain the spectra of noise-driven quasi-cycles. Using this theory we then present our main results in the section titled “Consequences of heterogeneity”. We investigate in detail how the heterogeneity in the population affects the properties of stochastic outbreaks of the disease. Finally, in the last section we summarize our findings.

## Model

We use an extension of the standard SIR model^48^, in a population of fixed size *N*. Broadly, each individual can be of one of three types: susceptible (S), infective (I) or recovered (R). The spreading of the disease is described by infection events. These occur either through contact of a susceptible with an infective individual, as described below, or through spontaneous infection. Individuals recover at rate *ρ*, and they die at rate *κ*. The death rate is assumed to be independent of the disease status of an individual. To keep the number of individuals in the population constant, any death event is immediately followed by a birth of a new susceptible individual. This is of course an assumption, valid for large enough populations, so that fluctuations in the overall size can be neglected. The assumption is mainly made for simplicity and is not uncommon (see e.g refs^16,49,50^).

We introduce heterogeneity by dividing the groups of susceptibles and infectives into subclasses. We will write *S*_*i*_ and *I*_*a*_ for these, with $i=1,\ldots ,K$ and $a=1,\ldots ,M$. Individuals in subgroup *S*_*i*_ have susceptibility ${\chi }_{i}$ to the disease, and infectives in class *I*_*a*_ have infectiousness ${\beta }_{a}$, which describes the propensity of the infective to pass on the disease to susceptible individuals. We write ${n}_{i}$ for the number of individuals of type *S*_*i*_, and ${m}_{a}$ for the number of individuals in class ${I}_{a}$.

**Figure 1:**
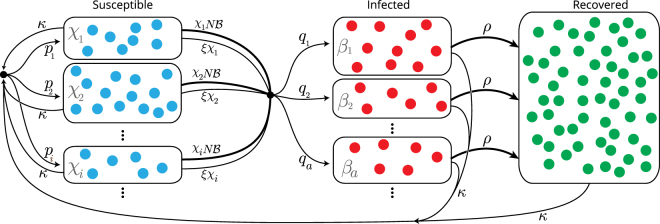
SIR model with heterogeneous susceptibility and infectivity. The diagram illustrates the different processes described by the model. New (susceptible) individuals are born at a rate *κ*, and they are assigned a susceptibility of *χ*_*i*_ with probability *p*_*i*_. Susceptible individuals transition to an infected state either by spontaneous infection or by contact with any of the infected classes. The former process occurs with rate *ξχ*_*i*_, if the susceptible is of type *S*_*i*_. Conact infection occurs at a rate ${\chi }_{i}N {\mathcal B}$, where $N {\mathcal B}$ is the total infective power of the population (see Eq. (3)). Once infected, the individual is assigned an infectiousness *β*_*a*_ with probability *q*_*a*_. All infected individuals recover at the same rate *ρ*. At any stage, individuals die with a rate *κ*. To keep the total population *N* constant, deceased individuals are immediately replaced by a new susceptible individual.

The dynamics are illustrated in Fig. 1, and can be summarised in the following reaction scheme:

1 $$\begin{array}{c}{\rm{Spontaneous}}\,\mathrm{infection}:\,{S}_{i}\mathop{\to }\limits^{\xi {\chi }_{i}{q}_{a}}{I}_{a}\,\\ \,\,\,\,\,{\rm{Infection}}\,{\rm{by}}\,\mathrm{contact}:\,\quad \,{S}_{i}+{I}_{a}\mathop{\to }\limits^{{\beta }_{a}{\chi }_{i}{q}_{b}}{I}_{a}+{I}_{b}\,\\ \quad \quad \quad \quad \,\,\,\,\mathrm{Recovery}:\,\,\,\,\,\,\,\,\,{I}_{a}\mathop{\to }\limits^{\rho }R\\ \quad \quad \quad \,\,\,\,\,\mathrm{Birth}/\mathrm{Death}:\,\quad \,\,\,\,\,\,\,\,\,{S}_{j}\mathop{\to }\limits^{{p}_{i}\kappa }{S}_{i}\\ \phantom{\rule{5em}{0ex}}\,\,\,\,\quad \quad \quad \quad \,\,\,\quad \,\,\,\,\,\,{I}_{a}\mathop{\to }\limits^{{p}_{i}\kappa }{S}_{i}\\ \phantom{\rule{5em}{0ex}}\,\,\,\,\quad \quad \quad \quad \,\,\,\,\quad \,\,\,\,\,R\mathop{\to }\limits^{{p}_{i}\kappa }{S}_{i},\end{array}$$

where $\{{p}_{i}\}$ and $\{{q}_{a}\}$ represent the probabilities of being assigned a susceptibility ${\chi }_{i}$ or infectiousness ${\beta }_{a}$ at birth or upon infection, respectively. The first of these reactions describes spontaneous infection, converting an individual in class *S*_*i*_ into an individual of type *I*_*a*_. The per-capita rate of events of this type is $\xi {\chi }_{i}{q}_{a}$, where $\xi$ is an overall inverse time scale for spontaneous infection, ${\chi }_{i}$ is the susceptibility of ${S}_{i}$ to the disease, and ${q}_{a}$ is the probability that the newly infected individual is in class *I*_*a*_. Similarly, the second reaction describes infection of an individual of type *S*_*i*_ upon contact with an individual of type *I*_*a*_. The newly infected individual is in class *I*_*b*_. Events of this particular type occur with a rate proportional to ${\beta }_{a}$ (the propensity of *I*_*a*_ to spread the disease), to ${\chi }_{i}$ (the susceptibility of *S*_*i*_) and to ${q}_{b}$. The third reaction describes recovery, and the final three reactions are birth/death events. The newly born individual is assumed to be randomly placed into one of the classes *S*_*i*_ ($i=1,\ldots ,K$), occurring with respective probability ${p}_{i}$. We note that our model does not describe potential correlations between the susceptibility of an individual and its infectivity after they become infected; our focus is on heterogeneity of susceptibility due to physiological factors, and not primarily due to contact patterns. Extensions to include correlations can however be constructed among similar lines.

The model defines a continuous-time Markov process, and can be simulated straightforwardly using for example the celebrated Gillespie algorithm^51^. The starting point for the analytical study of the model is the master equation. Our analysis below will be based on approximating the solution to this master equation by performing a system-size expansion^46^ and linear-noise approximation, leading to a stochastic differential equation describing the dynamics in the limit of large, but finite population size. In order to do this, it is useful to first introduce

2 $$\overline{\chi }=\sum _{i}{p}_{i}{\chi }_{i},\quad {\rm{and}}\quad {\mathfrak{X}}=\frac{1}{N}\sum _{i}{\chi }_{i}{n}_{i}\mathrm{.}$$

The quantity $\overline{\chi }$ is the mean susceptibility of a newly born individual, whereas $N{\mathfrak{X}}$ describes the aggregate susceptibility of the population. Similarly, we define

3 $$\overline{\beta }=\sum _{a}{q}_{a}{\beta }_{a}\quad {\rm{and}}\quad  {\mathcal B} =\frac{1}{N}\sum _{a}{\beta }_{a}{m}_{a},$$

where $\overline{\beta }$ represents the mean infectivity of a newly infected individual, and $N {\mathcal B}$ the total ‘infective power’ in the population. We note that $\overline{\chi }$ and $\overline{\beta }$ are fixed in time, and are properties of the distributions $\{{p}_{i},{\chi }_{i}\}$ and $\{{q}_{a},{\beta }_{i}\}$. The quantities ${\mathfrak{X}}$ and ${\mathcal B}$, on the other hand, are time-dependent and evolve as the composition of the population changes.

## Deterministic analysis

### Dynamics

In the limit of an infinite population, the dynamics can be described by deterministic equations for the quantities ${x}_{i}={\mathrm{lim}}_{N\to \infty }{n}_{i}/N$ and ${y}_{a}={\mathrm{lim}}_{N\to \infty }{m}_{a}/N$. They are given by

4 $$\begin{array}{c}{\dot{x}}_{i}=\kappa {p}_{i}-\kappa {x}_{i}-\xi {\chi }_{i}{x}_{i}-{\chi }_{i}{x}_{i} {\mathcal B} ,\\ {\dot{y}}_{a}=\xi {q}_{a}{\mathfrak{X}}+{q}_{a}{\mathfrak{X}} {\mathcal B} -\rho {y}_{a}-\kappa {y}_{a}\mathrm{.}\end{array}$$

These ordinary differential equations can be derived either by using direct mass-action kinetics, or from the lowest-order expressions in an expansion of the master equation in the inverse system size^46^.

Ultimately we will mostly be interested in aggregate quantities, i.e. the total density of susceptibles or infectives in the population, irrespective of what subclass they belong to. We therefore introduce

5 $$S=\sum _{i}{x}_{i}\quad {\rm{and}}\quad I=\sum _{a}{y}_{a}\mathrm{.}$$

From Eqs (4) we find

$$\dot{S}=\kappa -\kappa S-\xi {\mathfrak{X}}- {\mathcal B} {\mathfrak{X}},$$

6 $$\dot{I}=\xi {\mathfrak{X}}+{\mathfrak{X}} {\mathcal B} -\rho I-\kappa I\mathrm{.}$$

This system is not closed due to the presence of ${\mathfrak{X}}$ and ${\mathcal B}$ on the right-hand side. These quantities in turn evolve in time according to

7 $$\begin{array}{c}\dot{{\mathfrak{X}}}=\kappa \overline{\chi }-\kappa {\mathfrak{X}}-(\xi + {\mathcal B} )\sum _{i}{\chi }_{i}^{2}{x}_{i},\\ \dot{ {\mathcal B} }=\xi {\mathfrak{X}}\overline{\beta }+\overline{\beta }{\mathfrak{X}} {\mathcal B} -(\rho +\kappa ) {\mathcal B} ,\end{array}$$

which again does not close the set of equations, due to the presence of the term ${{\mathfrak{X}}}_{2}(t)\equiv {\sum }_{i}{\chi }_{i}^{2}{x}_{i}(t)$. Modulo normalisation, and recalling that the $\{{x}_{i}\}$ are time-dependent, this object is recognised as the second moment of the distribution of susceptibilities among the group of susceptibles *at time t*. It cannot be determined from Eqs (6) and (7) alone. Instead we find

8 $${\dot{{\mathfrak{X}}}}_{n}=\kappa \overline{{\chi }^{n}}-\kappa {{\mathfrak{X}}}_{n}-(\xi + {\mathcal B} ){{\mathfrak{X}}}_{n+1},$$

where we have introduced $\overline{{\chi }^{n}}={\sum }_{i}{p}_{i}{\chi }_{i}^{n}$ and ${{\mathfrak{X}}}_{n}={\sum }_{i}{x}_{i}{\chi }_{i}^{n}$. This indicates that the deterministic dynamics at the aggregate level is described by an infinite hierarchy of equations. This set of equations does not close in the transient regime. However, as we will see next, closure can be achieved assuming the system settles down to a fixed point in the long run.

### Fixed point

We proceed by a brief analysis of the fixed points of the deterministic dynamics. We will label these by a star. They can be obtained by setting ${\dot{x}}_{i}=0$ and ${\dot{y}}_{a}=0$ in Eq. (4), leading to

9 $$\begin{array}{c}{x}_{i}^{\star }=\frac{\kappa {p}_{i}}{\kappa +(\xi +{{\mathcal{B}}}^{\star }){\chi }_{i}},\\ {y}_{a}^{\star }=\frac{(\xi +{{\mathcal{B}}}^{\star }){{\mathfrak{X}}}^{\star }{q}_{a}}{\rho +\kappa }.\end{array}$$

Similarly, we find the fixed points of the aggregate quantities *S*, *I*, ${\mathfrak{X}}$ and ${\mathcal B}$ from Eqs (6,7). After re-arranging and using Eq. (9) we arrive at

10 $$\begin{array}{ccc}{S}^{\star } & = & 1-\frac{(\rho +\kappa )}{\kappa }\frac{{{\mathcal{B}}}^{\star }}{\bar{\beta }},\\ {I}^{\star } & = & \frac{{{\mathcal{B}}}^{\star }}{\bar{\beta }},\\ {{\mathfrak{X}}}^{\star } & = & \frac{(\rho +\kappa )}{(\xi +{{\mathcal{B}}}^{\star })}\frac{{{\mathcal{B}}}^{\star }}{\bar{\beta }},\\ {{\mathcal{B}}}^{\star } & = & \frac{\bar{\beta }\kappa }{(\rho +\kappa )}\sum _{i}(\frac{{\chi }_{i}{p}_{i}}{\frac{\kappa }{\xi +{{\mathcal{B}}}^{\star }}+{\chi }_{i}}),\end{array}$$

which is a closed set of equations, for a given set of model parameters $\{{p}_{i},{\chi }_{i},{q}_{a},{\beta }_{a}\}$.

We highlight that while the transient dynamics of the system described in terms of the four macroscopic variables *S*, *I*, ${\mathfrak{X}}$ and ${\mathcal B}$ generates an infinite hierarchy of equations, potential fixed points can be uniquely described by a closed set of equations, assuming that the distribution of susceptibilities at birth and of the propensity of newly infected individuals to pass on the disease are known. In other words, the fixed point can be obtained in terms of the model parameters $\{{q}_{a},{\beta }_{a}\}$ and $\{{p}_{i},{\chi }_{i}\}$. While we cannot provide an analytical proof that the deterministic system will always converge to a fixed point, we note that, for the range of parameter used, we have not detected a single case in which numerically integrating Eq. (4) did not lead to a fixed point. In this context it is useful to point out that, in a homogeneous model, any combination of susceptibility and infectivity within the range of parameters used here would lead to a basic reproductive number above unity. For such models it is known that stable fixed points are eventually reached^52^.

## Linear-noise approximation

We now proceed to analyse the effects of stochasticity in the model, with a particular focus on the interaction between heterogeneity of individuals in the population and the noise induced by the demographics of the finite system.

**Figure 2:**
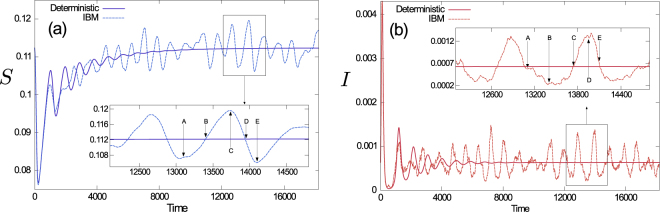
Population dynamics. Time series of the population density of total susceptible (panel (a)) and total infected individuals (panel (b)). Noise-sustained oscillations are clearly seen. The insets show a zoom in on the cycles. Labels $A,B,\ldots ,E$ are for later purposes (see below).

We illustrate these effects in Fig. 2, and show an example of both the deterministic time-evolution of the system (thick continuous lines) and a realization of an individual-based simulation (thin dashed lines); the latter illustrates the intrinsic stochasticity of the process. Even after the deterministic model has reached a fixed point, the individual-based model shows sustained oscillations around it. These oscillations arise from a combination of complex eigenvalues of the underlying deterministic dynamics and the presence of intrinsic noise coming from the Poissonian jump process of the master equation. We will focus our attention on these stochasticity-driven periodic outbreaks in the remainder of this article, and build on the mathematical analysis via the linear-noise approximation^20^. In particular we will study how the heterogeneity in the population affects the properties of these cycles.

### Stochastic Dynamics

In order to carry out an analysis of the stochastic dynamics, we write ${n}_{i}/N={x}_{i}+{\tilde{x}}_{i}/\sqrt{N}$, and ${m}_{a}/N={y}_{a}+{\tilde{y}}_{a}/\sqrt{N}$, where ${x}_{i}(t)$ and ${y}_{a}(t)$ are the solutions of the deterministic equations (4) and the quantities with a tilde describe the stochastic fluctuations about the deterministic trajectory. The above ansatz reflects the anticipation that these fluctuatons will have a relative magnitude of order ${N}^{-\mathrm{1/2}}$. We then carry out an expansion in the inverse system size up to and including sub-leading order^46^. In the fixed point regime of the deterministic dynamics we then arrive at

11 $$\begin{array}{c}{\dot{\mathop{x}\limits^{ \sim }}}_{i}=-\kappa {\mathop{x}\limits^{ \sim }}_{i}-(\xi +{{\mathcal{B}}}^{\star }){\chi }_{i}{\mathop{x}\limits^{ \sim }}_{i}-{\chi }_{i}{x}_{i}^{\star }\mathop{{\mathcal{B}}}\limits^{ \sim }+{\eta }_{i},\\ {\dot{\mathop{y}\limits^{ \sim }}}_{a}={q}_{a}(\xi \mathop{{\mathfrak{X}}}\limits^{ \sim }+\mathop{{\mathfrak{X}}}\limits^{ \sim }{{\mathcal{B}}}^{\star }+{{\mathfrak{X}}}^{\star }\mathop{{\mathcal{B}}}\limits^{ \sim })-(\rho +\kappa ){\mathop{y}\limits^{ \sim }}_{a}+{\nu }_{a}.\end{array}$$

The linear-noise approximation also applies during transients. All objects on the right-hand side of Eq. (11) then become time dependent. Since we ultimately focus on the oscillations about deterministic fixed point, we have not made this more explicit. The $\{{\eta }_{i}\}$ and $\{{\nu }_{a}\}$ are Gaussian white noise variables, with variance and co-variance (across components) as described in more detail in the Supplement (see Sec. S1). Writing $\tilde{S}={\sum }_{i}{\tilde{x}}_{i}$ and $\tilde{I}={\sum }_{a}{\tilde{y}}_{a}$ we find the following dynamics of fluctuations at the aggregate level,

12 $$\begin{array}{ccc}\dot{\mathop{S}\limits^{ \sim }} & = & -\kappa \mathop{S}\limits^{ \sim }-(\xi +{{\mathcal{B}}}^{\star })\mathop{{\mathfrak{X}}}\limits^{ \sim }-{{\mathfrak{X}}}^{\star }\mathop{{\mathcal{B}}}\limits^{ \sim }+\sum _{i}{\eta }_{i},\\ \dot{\mathop{I}\limits^{ \sim }} & = & (\xi +{{\mathcal{B}}}^{\star })\mathop{{\mathfrak{X}}}\limits^{ \sim }+{{\mathfrak{X}}}^{\star }\mathop{{\mathcal{B}}}\limits^{ \sim }-(\rho +\kappa )\mathop{I}\limits^{ \sim }+\sum _{a}{\nu }_{a},\\ \dot{\mathop{{\mathfrak{X}}}\limits^{ \sim }} & = & -\kappa \mathop{{\mathfrak{X}}}\limits^{ \sim }-{{\mathfrak{X}}}_{2}^{\star }\mathop{{\mathcal{B}}}\limits^{ \sim }-(\xi +{{\mathcal{B}}}^{\star })\sum _{i}{\chi }_{i}^{2}{\mathop{x}\limits^{ \sim }}_{i}+\sum _{i}{\chi }_{i}{\eta }_{i},\\ \dot{\mathop{{\mathcal{B}}}\limits^{ \sim }} & = & (\xi +{{\mathcal{B}}}^{\star })\bar{\beta }\mathop{{\mathfrak{X}}}\limits^{ \sim }+\bar{\beta }{{\mathfrak{X}}}^{\star }\mathop{{\mathcal{B}}}\limits^{ \sim }-(\rho +\kappa )\mathop{{\mathcal{B}}}\limits^{ \sim }+\sum _{a}{\beta }_{a}{\nu }_{a}.\end{array}$$

As in the deterministic analysis, this set of equations is not closed. It describes the dynamics of fluctuations about the deterministic fixed point, but makes no assumption of stationarity of the fluctuations (for example, correlation functions need not be time translation invariant). The lack of closure is due to the term ${\sum }_{i}{\chi }_{i}^{2}{\tilde{x}}_{i}$ in the equation for $\dot{\tilde{{\mathfrak{X}}}}$. However, we will show below that a closed set of equations in the stationary state (of fluctuations) can be derived.

### Fluctuation around the deterministic fixed point

We here show that although Eqs (12) are not closed, we can explore noise-induced oscillations around the deterministic fixed point. To this end, we introduce the Fourier transforms (with respect to time) of the variables ${\tilde{x}}_{i}$ and ${\tilde{y}}_{a}$. We will denote these by ${\widehat{x}}_{i}$ and ${\widehat{y}}_{a}$. From the Langevin equations (11) we find, after re-arranging,

13 $$\begin{array}{c}{\hat{x}}_{i}=\frac{-{\chi }_{i}{x}_{i}^{\star }\hat{{\mathcal{B}}}+{\hat{\eta }}_{i}}{i\omega +\kappa +(\xi +{{\mathcal{B}}}^{\star }){\chi }_{i}},\\ {\hat{y}}_{a}=\frac{[(\xi +{{\mathcal{B}}}^{\star })\hat{{\mathfrak{X}}}+{{\mathfrak{X}}}^{\star }\hat{{\mathcal{B}}}]{q}_{a}+{\hat{\nu }}_{a}}{i\omega +\rho +\kappa }.\end{array}$$

The noise variables $\{{\eta }_{i}\}$ and $\{{\nu }_{a}\}$ are uncorrelated in time, and their variance and correlation across components can be expressed in terms of known quantities (see Eq. (S3) in the Supplement). The variable *ω* is the conjugate of time under Fourier transform. Similarly, we find the following for the relevant aggregate quantities,

14 $$\begin{array}{rcl}\widehat{S} & = & \frac{1}{i\omega +\kappa }[-\frac{i\omega +D}{\overline{\beta }}\widehat{ {\mathcal B} }+\frac{1}{\overline{\beta }}\sum _{a}{\beta }_{a}{\widehat{\nu }}_{a}+\sum _{i}{\widehat{\eta }}_{i}],\\ \widehat{I} & = & \frac{1}{i\omega +D}[\frac{i\omega +D}{\overline{\beta }}\widehat{ {\mathcal B} }-\frac{1}{\overline{\beta }}\sum _{a}{\beta }_{a}{\widehat{\nu }}_{a}+\sum _{a}{\widehat{\nu }}_{a}],\\ \mathop{{\mathfrak{X}}}\limits^{\frown {}} & = & \frac{1}{\overline{\beta }C}[(i\omega +E)\widehat{ {\mathcal B} }-\sum _{a}{\beta }_{a}{\widehat{\nu }}_{a}],\\ \widehat{ {\mathcal B} } & = & \frac{\overline{\beta }C\sum _{i}\frac{{\chi }_{i}{\widehat{\eta }}_{i}}{i\omega +{A}_{i}}+\sum _{a}{\beta }_{a}{\widehat{\nu }}_{a}}{i\omega +E+\overline{\beta }C\kappa \sum _{i}\frac{{\chi }_{i}^{2}{p}_{i}}{{A}_{i}(i\omega +{A}_{i})}},\end{array}$$

where, for simplicity, we have introduced the notation

15 $$\begin{array}{ccc}{A}_{i} & = & \kappa +(\xi +{{\mathcal{B}}}^{\star }){\chi }_{i},\\ C & = & \xi +{{\mathcal{B}}}^{\star },\\ D & = & \rho +\kappa ,\\ E & = & \rho +\kappa -\bar{\beta }{{\mathfrak{X}}}^{\star }.\end{array}$$

Equations (14) constitute a closed set of equations for the Fourier transforms of the aggregate fluctuations $\tilde{S},\tilde{I},\tilde{{\mathfrak{X}}}$ and $\tilde{ {\mathcal B} }$ in the stationary state. We thus make an observation similar to that in the deterministic analysis: although we cannot describe the evolution of fluctuations in the transient regime, we can derive a closed description of the statistics of fluctuations about deterministic fixed points within the linear-noise approximation.

### Power Spectral Density

Equation (14) can be used to describe the periodic cycles shown in Fig. 2; we will now proceed to analyse these in more detail. Specifically, we will use the above results to compute the power spectral density (PSD) of fluctuations. This allows us to identify the characteristic frequency of noise-driven epidemic cycles, and to infer information about their amplitude.

The (average) power spectral density of a time series, $z(t)$, generated from the stochastic individual-based model, is given by ${{\mathcal{P}}}_{z}(\omega )=\langle |\widehat{z}(\omega {)|}^{2}\rangle$, where $\langle \cdots \rangle$ stands for an average over realizations of the stochastic dynamics. The PSD can be computed analytically for all individual signals *x*_*i*_, *y*_*a*_, and for the aggregate variables *S*, *I*, ${\mathfrak{X}}$ and ${\mathcal B}$. The resulting expressions are lengthy; for completeness we provide them in the Supplement (see Sec. S2). As an illustration we here show the PSD of ${\mathcal B}$,

16 $${{\mathcal{P}}}_{{\mathcal{B}}}(\omega )=\frac{2{{\mathfrak{X}}}^{\star }C}{|g{|}^{2}}(\bar{{\beta }^{2}}-\frac{{\bar{\beta }}^{2}C\kappa }{D}\sum _{i}\frac{{\chi }_{i}{p}_{i}{A}_{i}}{{\omega }^{2}+{A}_{i}^{2}})-\frac{{(\bar{\beta }C\kappa )}^{2}}{|g{|}^{2}}[\sum _{i,j}\frac{{p}_{i}{p}_{j}{\chi }_{i}{\chi }_{j}({A}_{i}+{A}_{j})({\omega }^{2}+{A}_{i}{A}_{j})}{{A}_{i}{A}_{j}({\omega }^{2}+{A}_{i}^{2})({\omega }^{2}+{A}_{j}^{2})}],$$

with

17 $$|g{|}^{2}={[E+\overline{\beta }C\kappa \sum _{i}\frac{{\chi }_{i}^{2}{p}_{i}}{{\omega }^{2}+{A}_{i}^{2}}]}^{2}+{\omega }^{2}{[1-\overline{\beta }C\kappa \sum _{i}\frac{{\chi }_{i}^{2}{p}_{i}}{{A}_{i}({\omega }^{2}+{A}_{i}^{2})}]}^{2}\mathrm{.}$$

As detailed in the Supplement (see Sec. S2) the power spectra of *S*, *I* and ${\mathfrak{X}}$ can be expressed in terms of that of ${\mathcal B}$; many of the characteristics of the spectra of $S,I$ and ${\mathfrak{X}}$ are shared with those of ${\mathcal B}$, or directly related to it. We note that the RHS of Eq. (16) is proportional to $\mathrm{1/|}g{|}^{2}$, and the same is the case for the spectral densities of ${\mathfrak{X}},S$ and *I* (see Eq. (S10) in the Supplement); as a result, some of the key properties of the power spectra are determined by the behaviour of $|g{|}^{2}$, as discussed in more detail below.

### Test Against Simulations

**Figure 3:**
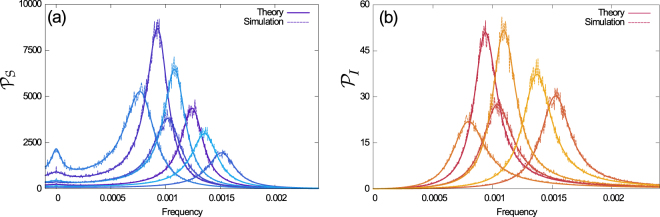
Power spectral densities of the fluctuations of **(a)** Susceptible and **(b)** Infected population for seven different examples of the model, generated as explained in more detail in the text. In all cases theory and simulations agree.

To illustrate the model and test our analytical results, we sampled possible heterogeneous populations. Specifically, the simulations shown in Fig. 3 are for populations with five susceptible and three infected subclasses. For each example, the probabilities $\{{p}_{i}\}$ and $\{{q}_{a}\}$ were drawn at random from a flat distribution over the simplexes ${\sum }_{i}{p}_{i}=1$ and ${\sum }_{a}{q}_{a}=1$. Susceptibilities and infectivities were assigned randomly in the intervals $0.5\le {\chi }_{i}\le 2.5$ and $0.3\le {\beta }_{a}\le 1.3$. Simulations are for *N* = 10^6^, and the rates for recovery, birth/death and immigration were set at *ρ* = 0.07, $\kappa =5.5\times {10}^{-5}$ and $\xi =5\times {10}^{-6}$ respectively. The rates *β*_a_, *ρ*, *κ* and $\xi$ have units of days^−1^, whereas ${\chi }_{i}$ is dimensionless. The chosen rates are representative of childhood diseases such as whooping cough, measles, rubella or chickenpox^53^.

The resulting PSDs are shown in Fig. 3. The continuous thick lines show the analytical result, and dashed lines are obtained from simulations, as an average over realizations of the individual-based model. As can be seen from the figure, the predictions of Eq. (S10) precisely match the results from simulations. In all figures, axes labelled ‘frequency’ show $f=\omega \mathrm{/2}\pi$, and have units of days^−1^.

It is interesting to note that the power spectral density can remain non-zero at zero frequency. A more detailed analysis reveals that its value is finite (i.e., not diverging), there is no evidence of e.g. a delta-peak at *ω* = 0. This indicates that the area under the overall correlation function of fluctuations is non-zero, but finite, and there is no discernible shift of the overall stationary equilibrium (such a shift would result in a diverging contribution to the power spectrum at *ω* = 0).

## Consequences of Heterogeneity

Having established an analytical description of quasi-cycles, we now use this theory to identify which properties of the distribution of *p*_*i*_, *χ*_*i*_, *q*_*a*_ and *β*_*a*_ are most relevant for the characteristics of stochastic quasi-cycles in heterogeneous populations. Specifically, we study how heterogeneity in the population affects the dominant frequency of quasi-cycles, their amplitude and the sharpness of the spectra. We will then also discuss if and how the different subgroups synchronise during the epidemic cycles.

### Dominant Cycle Frequency

Numerical inspection of the different terms in the analytical solution of the PSDs suggests that the dominating element is the factor $\mathrm{1/|}g{|}^{2}$, as briefly indicated above. The frequency for which $|g{|}^{2}$ reaches its minimum roughly corresponds to the dominant cycle frequency, *ω*_*d*_, in the PSDs. The minimum of $|g{|}^{2}$ can be found by differentiation of the expression in Eq. (17). Assuming that $C{\chi }_{i}\gg \kappa$, we further approximate the location of this minimum. This assumption is valid if infection processes occur on a time scale which is much shorter than the life expectancy of an individual. Further, we assume that $\omega \gg {A}_{i}$, i.e., that a susceptible individual typically lives through several epidemic events before it becomes infected. Both approximations are intuitively plausible for childhood diseases, known to show periodic outbreaks^53^. Making these assumptions we find that the frequency for which $|g{|}^{2}$ is minimal can be approximated as

18 $${\omega }_{d}\approx \sqrt{\kappa \overline{\chi }\overline{\beta }}\mathrm{.}$$

This implies that the characteristic frequency is determined (mostly) by the mean susceptibility at birth and the mean infectivity at infection ($\overline{\chi }$ and $\overline{\beta }$) and the capacity of replenishment of the susceptible pool (*κ*).

**Figure 4:**
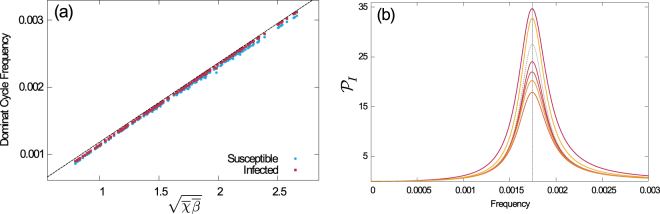
Verification of approximation (18) for the dominating frequency of cycles. **(a)** Frequency $f=\omega /2\pi$ at the maximum of the PSD, determined from Eq. (S10) as a function of $\sqrt{\overline{\chi }\overline{\beta }}$, for fixed $\kappa$. The black dashed line corresponds to Eq. (18). Markers are from 200 different populations, each with 5 susceptible and 3 infected subgroups, and with random choices of $\{{p}_{i},{\chi }_{i},{q}_{a},{\beta }_{a}\}$. The values of *χ*_*i*_ and *β*_*a*_ were chosen from the interval 1.7±1.6999995; *q*_*a*_ and *p*_*i*_ from a flat distribution. This resulted in values of $\overline{\chi }$ and $\overline{\beta }$ in the range 0.3 to 3.3, and for $\overline{{\chi }^{2}}$ and $\overline{{\beta }^{2}}$ in the range 0.1 to 10. **(b)** PSD of the total infected population of different random distributions of $\{{p}_{i},{\chi }_{i},{q}_{a},{\beta }_{a}\}$, with equal values for $\overline{\chi }$ and $\overline{\beta }$, but different values of $\overline{{\chi }^{2}}$ and $\overline{{\beta }^{2}}$. As a consequence of Eqs (18) and (19), the characteristic frequency is the same for all such samples, but the height of the peak in the PSD varies considerably (the amplitude of the oscillations changes with the square root of the amplitude of the power spectra). The dashed grey line corresponds to the homogeneous model, i.e. *K* = M = 1. The vertical dotted line is a visual aid.

The validity of our approach is confirmed in Fig. 4(a), where we test the approximation against simulations for a wide set of parameters. A perhaps more intuitive representation of our result can be found in Fig. 4(b), where we show the power spectra of several sample populations, each with different distributions of $\{{p}_{i},{\chi }_{i},{q}_{a},{\beta }_{a}\}$, but all with the same first moments $\overline{\chi }$ and $\overline{\beta }$. As seen in the figure, this produces spectra of different amplitudes but with the same characteristic frequency. For comparison we include the homogeneous case *K* = M = 1.

### Amplitude of Stochastic Cycles

While we have found above that the dominant frequency of stochastic cycles is largely determined by the first moments $\overline{\chi }$ and $\overline{\beta }$, the results shown in Fig. 4(b) demonstrate that this is not the case for the amplitude of the spectra at the dominant frequency. To investigate this further, we evaluate the analytic expressions for the PSDs in Eq. (S10) at the approximation of *ω*_*d*_ in Eq. (18). Making the same assumptions as in the previous section, we find that the height of the peak in the power spectra can be approximated as

19 $$\begin{array}{c}{{\mathcal{P}}}_{I}({\omega }_{d})\approx \frac{2(\rho +\kappa )}{{[\frac{(\rho +\kappa )\xi }{{{\mathcal{B}}}^{\star }}+\frac{{{\mathcal{B}}}^{\star }\bar{{\chi }^{2}}}{\bar{\chi }}]}^{2}}\frac{\bar{{\beta }^{2}}}{{\bar{\beta }}^{3}},\\ {{\mathcal{P}}}_{S}({\omega }_{d})\approx \frac{{(\rho +\kappa )}^{2}}{\kappa \bar{\chi }\bar{\beta }}{{\mathcal{P}}}_{I}({\omega }_{d}).\end{array}$$

We note the presence of the second moments $\overline{{\chi }^{2}}$ and $\overline{{\beta }^{2}}$, unlike in Eq. (18). This indicates that the spread of susceptibilities and infectivities is relevant to the size of the fluctuations about the endemic equilibrium. We note that the case *K* = M = 1 in Fig. 4(b) is special, as it leads to zero variance of the disorder by construction. We have experimented with the number of groups, *K* and *M*, and to a good approximation we find that the number of groups only affects the height and location of the peak in the spectrum through the mean and variance of the distributions of *β* and *χ*.

**Figure 5:**
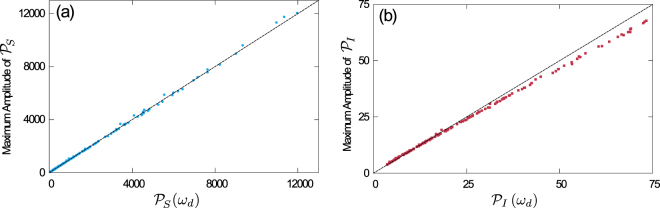
Verification of approximation (19) for the peak-height of the spectral densities. Horizontal axes show the prediction of Eq. (19) for susceptibles **(a)**, and infectives **(b)**. On the vertical axis we show the height at the peak of the spectra, as determined numerically from Eq. (S10) in the Supplement. Black dashed lines are the diagonal (‘*y* = *x*’), and markers represent the populations described in Fig. 4.

In Fig. 5 we plot results from the approximation in Eq. (19) against the maximum amplitude of spectra obtained numerically from the full expression (within the LNA), see Eq. (S10) in the Supplement. The data confirms that the approximation is valid for a wide range of parameters. While we find slight deviations at large amplitudes in the case of the infectives, the approximation is very robust for the susceptible population.

### Sharpness of the Spectra

We now turn to the sharpness of the peak in the PSDs. The sharper the peak, the closer the stochastic outbreaks are to perfect cyclic behaviour. Conversely, cyclic behaviour is less distinct if the peak in the spectrum is shallow. This has been described before as the ‘coherence’ of the spectra^16^. As we will investigate a different notion of coherence in the following section and in order to avoid confusion, we will refer to the concentration of power near the peak of the spectrum as ‘sharpness’.

Following^16^, we define the sharpness as the relative spectral power accumulated in an interval around the peak,

20 $${\mathbb{S}}=\frac{{\int }_{{\omega }_{d}-{\rm{\Delta }}\omega }^{{\omega }_{d}+{\rm{\Delta }}\omega }{\mathcal{P}}(\omega \mathrm{)\ }d\omega }{{\int }_{-\infty }^{+\infty }{\mathcal{P}}(\omega \mathrm{)\ }d\omega }\mathrm{.}$$

We compute the sharpness numerically, using the expressions in Eq. (S10). In order to evaluate the denominator in Eq. (20) we integrate up to an upper cutoff of ${\omega }_{max}=\pi /100\,\,{\rm{days}}{}^{-1}$. In the numerator we use ${\rm{\Delta }}\omega \,=\,0.05\,{\omega }_{max}$. The choice of ${\rm{\Delta }}\omega$ can be illustrated using Fig. 4(b), where the sharpness ${\mathbb{S}}$ of the peak roughly corresponds to the fraction of total power concentrated in the interval between frequencies of 0.0015 and 0.002 days^−1^.

**Figure 6:**
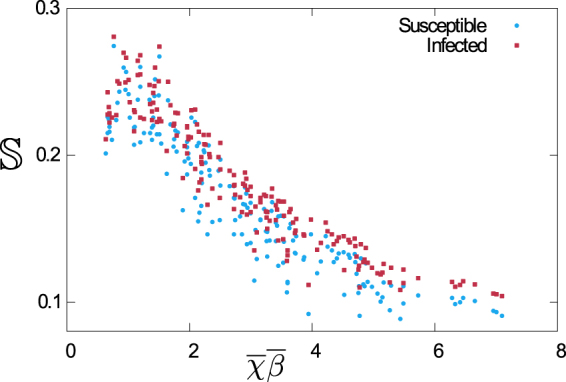
Sharpness of the power spectra as a function of the product of the mean susceptibilities and infectivities at birth/infection. Data is for the populations described in Fig. 4.

In Fig. 6 we show the sharpness of spectra for 200 random populations (as described in Fig. 4). It is clear from the figure that there is a trend of increasing sharpness as the product of the mean susceptibility and infectivity at birth approaches unity (in the dimensions used here). The spread of the markers on the vertical axis indicates that there are significant effects of heterogeneity. It proves difficult, though, to find a functional dependence on higher moments of the distributions of susceptibilities and/or infectivities which would further collapse the data. While we do not show this data here in detail, we have also experimented with heterogeneity drawn from several distributions (e.g. flat, normal, Gamma). Results suggest that–to a good approximation–the functional shape of the spectra is determined by $\overline{\beta },\overline{\chi },\overline{{\beta }^{2}}$ and $\overline{{\chi }^{2}}$, i.e. by the first two moments of the heterogeneity. Higher-order features do not seem to play an important role. We have also tested the stronger property of full collapse upon re-scaling by peak height and location of peak, i.e. whether there is a scaling property of the type $P(\omega )={P}_{{\rm{\max }}}\times f(\omega /{\omega }_{d})$. This appears not to be the case.

### Synchronization between Subgroups

**Figure 7:**
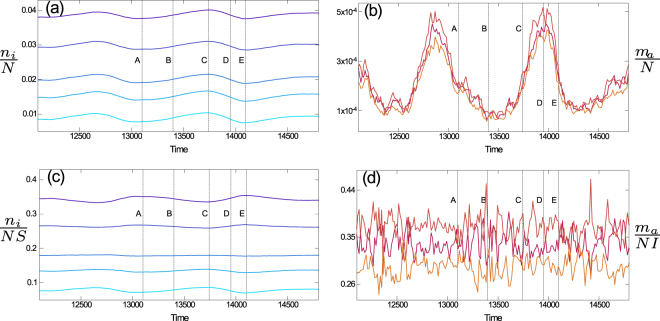
Stochastic cycles in subgroups of susceptibles and infectives. We show the same simulation run as in Fig. 2, but now split up into the different subgroups. Panels (a) and (b) show the number of individuals in each susceptible and infective subgroup normalised by the total population (*N*). In panels (c) and (d), we show the number of individuals in each subgroup divided by the total number of susceptible or infected individuals, respectively (*NS* and *NI*). Lines labelled *A* to *E* refer to points in the cycles of the aggregate variables *S*, *I* shown in Fig. 2.

We have established so far that introducing heterogeneity leads to significant changes in the quasi-cycles of the aggregate numbers of susceptible and infective individuals. However, we have not yet said much about the dynamics of the individual subgroups. In Fig. 7 we show the same example of sustained oscillations as in the inset of Fig. 2, but instead of the total susceptible and infected population we now highlight the time evolution of each of the subgroups.

**Figure 8:**
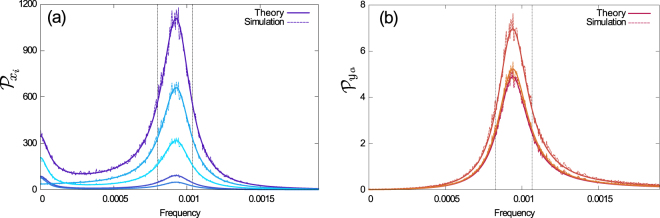
Power spectra of fluctuations for different subclasses of susceptibles and infectives. We use the same sample of the model parameters $\{{\chi }_{i},{p}_{i},{\beta }_{a},{q}_{a}\}$ as in Fig. 3. Simulations are averaged over multiple realizations of the stochastic dynamics, at fixed model parameters. The vertical dotted lines are for later purposes and mark the locations at which the power spectra take values approximately equal to half the maximum amplitude.

In the upper two panels, (a) and (b), we show time series of the number of individuals in each subgroup normalised by the total population size. More specifically, we show susceptible subclasses (${n}_{i}/N$) in panel (a), and infective subclasses (${m}_{a}/N$) in panel (b). For each of these, stochastic oscillations can be observed. These cycles are pronounced for the case of the infective subgroups, panel (b), and more shallow for the susceptibles, panel (a). This is to be expected, given that the total number of susceptibles is more than an order of magnitude larger than those of the infectives (see also Fig. 2). From Fig. 7(a), (b) it is clear that all subgroups undergo cycling of roughly the same frequency. This is confirmed by the power spectra in Fig. 8.

We note that these statements rely on expressing number of individuals in each class as a fraction of the total population, and not relative to the time-dependent total number of susceptibles or infectives, respectively. We contrast the above with a representation in which we express the occupancy in each infective subgroup as a fraction of the infectives only, and similarly for the susceptibles. To this end we replot the simulation run shown in Fig. 7(a), (b), but now in terms of ${n}_{i}/(NS)$ and ${m}_{a}/(NI)$, respectively. The quantities $NS={\sum }_{j}{n}_{j}$ and $NI={\sum }_{b}{m}_{b}$ are the total number susceptible and infective individuals, respectively, and they are time-dependent themselves. Results are shown in Fig. 7(c), (d). Although the overall number of infectives, *NI*, undergoes the noise-driven cycles shown in Fig. 2, we find no discernible structure within the group of infectives; the time series ${m}_{a}/(NI)$ in Fig. 7(d) are essentially flat noisy lines. This is what one would expect, since the allocation to each subgroup, *I*_*a*_, of infectives is random when an individual is newly infected, and the recovery rate is the same for all infective subgroups.

A more complex behaviour can be seen within the group of susceptibles. This group as a whole undergoes stochastic cycles (see Fig. 2), but an interesting structure is observed within the group of susceptibles as well. The time series ${n}_{i}/(NS)$ in Fig. 7(c) show cyclic behaviour, and–to a good approximation–any pair of these time series is either in phase with each other, or they have a phase difference of ±*π*.

To explore the phase lag between the different time series we use the so-called complex coherence function^54^. This technique relies on computing the cross-spectrum $\langle {\widehat{x}}_{i}(\omega ){\widehat{x}}_{j}^{\ast }(\omega )\rangle$ between time series ${x}_{i}(t)$ and ${x}_{j}(t)$. The phase lag is then obtained as

21 $${ {\mathcal L} }_{{x}_{i}{x}_{j}}(\omega )={\tan }^{-1}\frac{\,\text{Im}\,\langle {\widehat{x}}_{i}(\omega ){\widehat{x}}_{j}^{\ast }(\omega )\rangle }{\,\mathrm{Re}\,\langle {\widehat{x}}_{i}(\omega ){\widehat{x}}_{j}^{\ast }(\omega )\rangle }\mathrm{.}$$

We stress that the subscript * denotes complex conjugation, and is not to be confused with *, used earlier to indicate fixed points of the deterministic dynamics. Eq. (21) returns a phase lag for each spectral component, *ω*. Details can be found in the Supplement (see Sec. S3).

**Figure 9:**
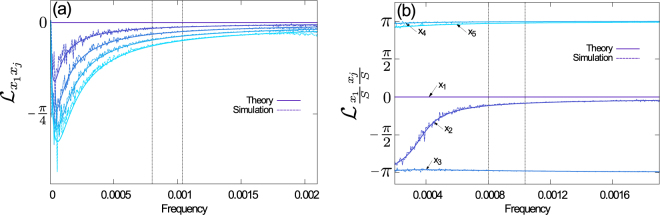
Phase-lag of time series between different subgroups of susceptibles. Data is for the same setup as in Fig. 7. We show the phase-lag between subgroups *i* and reference subgroup 1. Panel (a) depicts the case in which time series are normalized with respect to the total population, *N*; in panel (b) input time series are normalized with respect to the total number of susceptibles *NS*. As in Fig. 8, the vertical dotted lines mark the half-width of the peaks in the corresponding power spectra.

The phase lag between the different groups of susceptible individuals is shown in Fig. 9. The data in panel (a) corresponds to Fig. 7(a). More precisely, in Fig. 9(a) we pick the time series *n*_1_/*N* as a reference, and show the phase lag of all subgroups *n*_*i*_/*N* with respect to this reference time series. We find that the phase lag for frequencies around the dominant frequency in the power spectra is small, consistent with Fig. 7(a); all time series *n*_*i*_/*N* oscillate (roughly) in phase with each other. In Fig. 9(b) we repeat this procedure, but now taking the time series ${n}_{i}/(NS)$ as an input, corresponding to Fig. 7(c). One then finds a rather different picture; the phase lag around the dominant frequency takes values either near zero, or close to ±*π*. This indicates that the different classes of susceptible individuals fall into two groups. The time series in either group are in phase with each other, and in anti-phase with those in the respective other group. A closer inspection shows that these two groups are formed by the time series *i* with ${x}_{i}^{\star } < {S}^{\star }/K$ and with ${x}_{i}^{\star } > {S}^{\star }/K$ respectively. This behaviour in turn can be understood intuitively by revisiting Eq. (9). Assuming $\kappa \ll (\xi +{{\mathcal{B}}}^{\star }){\chi }_{i}$ for all *i* (a valid approximation for the cases analysed here), we find ${x}_{i}^{\star }\propto 1/{\chi }_{i}$, indicating that the more susceptible classes are less populated at the deterministic fixed point than the less susceptible ones. During the increasing leg of a stochastic cycle, we expect the number of newly infected individuals among class *i* to be proportional to ${x}_{i}^{\star }{\chi }_{i}$, suggesting that all susceptible classes are depleted in equal absolute numbers. This in turn means that subclasses with ${x}_{i}^{\star } > {S}^{\star }/K$ will represent an even larger fraction of the susceptible population as the total susceptible population decreases, while the subclasses with ${x}_{i}^{\star } < {S}^{\star }/K$ will represent a smaller fraction. This is what is observed in Fig. 7(c).

## Conclusions

In summary, we have explored the SIR model in finite populations, including demographic processes and allowed for agent-to-agent heterogeneity in both the susceptibility to a disease and the capacity to spread the disease. This system combines the effects of intrinsic demographic stochasticity (due to random infection, recovery and birth-death events), with quenched heterogeneity. The focus of our paper is to characterise the interplay between these two types of stochasticity, and to investigate how the heterogeneity between individuals affects quasi-cycles driven by intrinsic noise. Our analysis relies on the system-size expansion, which allows us to compute the properties of these cycles analytically in the linear-noise approximation.

Our principal results can be summarised as follows: (i) In the deterministic limit of infinite populations, no closed set of equations for macroscopic quantities can be found in the transient regime. Fixed points for aggregate quantities of this deterministic dynamics can however be fully determined from a set of closed equations for the total susceptible (S*) and infected (I*) population, and weighted averages of the susceptibility (${\mathfrak{X}}$*) and infectivity (${{\mathcal{B}}}^{\star }$). (ii) Similarly, the Langevin equations in the linear-noise approximation do not close easily at the aggregate level, but a closed set of equations for the spectra of fluctuations in $S,I,{\mathfrak{X}}$ and ${\mathcal B}$ about the deterministic fixed point can be found in the stationary state. These can be used to analytically describe the stochastic oscillations about the fixed point. (iii) Within reasonable assumptions, the characteristic frequency of the noise-driven oscillations is determined mostly by the mean susceptibility and infectivity at birth or infection ($\overline{\chi }$ and $\overline{\beta }$). However, the amplitude of the oscillations and the sharpness of peaks in the power spectra will generally depend on the higher moments of the distribution of susceptibilities and infectivities, in particular also on the agent-to-agent heterogeneity. (iv) Finally, the number of individuals in the different subclasses of infectives and susceptibles undergo stochastic cycles as well. If expressed in relation to the total population, these time series are synchronised and in phase. Normalized against the time-dependent total number of infectives, however, the different infective subclasses show no discernible oscillatory behaviour. Using a similar normalization within the susceptible population, we find that different subclasses are syncronized and either in phase with each other or have a phase difference of ±*π*. These results are confirmed analytically. Regardless of the normalization, we find that the periodic outbreaks do not follow a hierarchical infection process, and all subgroups have similar absolute depletion/increase in absolute numbers. This is in contrast to what has been reported in single outbreak studies^38,42^. However, it is important to note that in this existing work the outbreak is tracked in an initial transient period. Our results are valid after this period, at a deterministic fixed point, where the susceptible population is distributed in inverse proportion to their susceptibility (as explained above); this is a scenario different to the one studied in refs^38,42^.

We think our results can be relevant for future work in several ways. First, our work contributes to the ongoing discussion about when and how a model with heterogeneity can be replaced or approximated by a homogeneous model. In previous studies, heterogeneous models were compared to homogeneous models with susceptibility equivalent to the arithmetic^55^ or harmonic mean^44^ of the susceptibilities in the different groups. More recently, the focus has been placed on equivalent basic reproduction numbers (*R*_0_)^56^. In the heterogeneous model this requires estimating *R*_0_ based on, for example, the outbreak size, and therefore the comparison is not straightforward. Here we have shown that all models within the class we have looked at and with equal values of $\overline{\chi }\overline{\beta }$ generate periodic outbreaks with the same dominating frequency. This characteristic frequency can be used to define a unique homogeneous model to which models of varying degrees of heterogeneity can be compared. Furthermore, the dependence of the spectra of oscillations on both the first and higher moments of the distribution of heterogeneity might provide an avenue towards estimating how heterogeneous a population is from the observation of epidemic cycles. Finally, the formalism we have developed is versatile and can be applied to study quasi-cycles in other areas in which heterogeneity might be relevant, for example in predator-prey dynamics or evolution^20,22,57–60^. Our findings indicate that the frequency of quasi-cycles can, to a good approximation, be obtained from the first moment of the distribution of heterogeneous agent properties, but that their amplitude depends on higher moments of the disorder. We expect similar behaviour in other heterogeneous systems with noise-driven cycles.

## Electronic supplementary material

Supplementary Information
