# Supplementary material for: The effects of heterogeneity on stochastic cycles in epidemics
Source: Sci Rep. 2017 Oct 11;7:13008. doi: 10.1038/s41598-017-12606-x (PMC5636822; doi:10.1038/s41598-017-12606-x)
Supplement: Supplementary file 1 — Supplementary Information [file 41598_2017_12606_MOESM1_ESM.pdf]

The effects of heterogeneity on stochastic cycles in epidemics

Supplemental Material

Francisco Herrerías-Azcué and Tobias Galla
francisco.herreriasazcue@postgrad.manchester.ac.uk, tobias.galla@manchester.ac.uk

Theoretical Physics, School of Physics and Astronomy,
The University of Manchester, Manchester M13 9PL, United Kingdom

S1 Linear-noise approximation

Carrying out the system-size expansion for the model with heterogeneity is tedious, but straightforward and follows the lines of[1]. The final outcome is the linear-noise approximation in Eqs. (11). The variables η_i and ν_a, represent Gaussian noise, with no correlation in time, but with potential correlation between the different noise variables at equal time. These noise variables can be decomposed as

$$\begin{aligned}\eta_i &= -\sum_a u_{ia} - \sum_{ab} v_{iab} - \sum_{k\neq i} x_{ik} + \sum_{k\neq i} x_{ki} + \sum_a y_{ai} + z_i,\\ \nu_a &= \sum_i u_{ia} + \sum_{ib} v_{iba} - w_a - \sum_i y_{ai},\end{aligned} \tag{S1}$$

where, broadly speaking, each term on the right-hand side represents one possible type of event in the microscopic model. For example, u_{ia} relates to spontaneous infection of a susceptible individual of type S_i, resulting in a newly infective of type I_a. Similarly, v_{iab} represents an event in which an individual of type S_i is infected by an individual of type I_a, and the newly infected is of type I_b. The variable w_a relates to a recovery event of an individual of type I_a, death of susceptible S_i and simultaneous birth of susceptible S_k is reflected by x_{ik}; death of an individual of type I_a and simultaenous birth of susceptible S_i is described by y_{ai}, and finally death of a recovered individual and simultaneous birth of susceptible S_i, by z_i. The signs on the right-hand-side in Eqs. (S1) reflect the fact that each of these events may either increase or reduce the number of individuals of type S_i and I_a, respectively.

Each of the noise variables on the right-hand-side of Eqs. (S1) are uncorrelated in time, and they have no cross-correlations. Within the LNA their variances are set by the corresponding reaction rates at the deterministic fixed point, i.e. we have

$$\begin{aligned}\langle u_{ia}(t)u_{ia}(t')\rangle &= \xi\chi_i q_a x_i^\star \delta(t-t'),\\ \langle v_{iab}(t)v_{iab}(t')\rangle &= \beta_a\chi_i q_b x_i^\star I_a^\star \delta(t-t'),\\ \langle w_a(t)w_a(t')\rangle &= \rho I_a^\star\delta(t-t'),\\ \langle x_{ik}(t)x_{ik}(t')\rangle &= p_k\kappa x_i^\star\delta(t-t'),\\ \langle y_{ai}(t)y_{ai}(t')\rangle &= p_i\kappa I_a^\star\delta(t-t'),\\ \langle z_i(t)z_i(t')\rangle &= (1-S^\star-I^\star)p_i\kappa\delta(t-t').\end{aligned} \tag{S2}$$

Using the shorthand introduced in Eqs. (15), we then find

$$\begin{aligned}\langle \eta_i(t)\eta_j(t')\rangle &= -\kappa^2\left(\frac{1}{A_i}+\frac{1}{A_j}\right)p_ip_j\delta(t-t'),\ \text{for } i\neq j,\\ \langle \eta_i(t)\eta_i(t')\rangle &= 2\kappa\left(1-\frac{\kappa p_i}{A_i}\right)p_i\delta(t-t'),\\ \langle \nu_a(t)\nu_b(t')\rangle &= 0,\ \text{for } a\neq b\\ \langle \nu_a(t)\nu_a(t')\rangle &= 2C\mathfrak{X}^\star q_a\delta(t-t'),\\ \langle \eta_i(t)\nu_a(t')\rangle &= -\kappa C\left(\frac{\chi_i}{A_i}+\frac{\mathfrak{X}^\star}{D}\right)p_iq_a\delta(t-t'),\end{aligned} \tag{S3}$$

which are needed for the computation of the PSDs.

S2 Calculation of power spectra

We start from the result in Eqs. (14) in Sect. 3.2:

$$
\begin{aligned}
\widehat{S}(\omega) &= \frac{1}{i\omega+\kappa}\left[-\frac{i\omega+D}{\overline{\beta}}\widehat{\mathcal{B}}+\frac{1}{\overline{\beta}}\sum_a \beta_a\widehat{\nu}_a+\sum_i \widehat{\eta}_i\right],\\
\widehat{I}(\omega) &= \frac{1}{i\omega+D}\left[\frac{i\omega+D}{\overline{\beta}}\widehat{\mathcal{B}}-\frac{1}{\overline{\beta}}\sum_a \beta_a\widehat{\nu}_a+\sum_a \widehat{\nu}_a\right],\\
\widehat{\mathfrak{X}}(\omega) &= \frac{1}{\overline{\beta}C}\left[(i\omega+E)\,\widehat{\mathcal{B}}-\sum_a \beta_a\widehat{\nu}_a\right],\\
\widehat{\mathcal{B}}(\omega) &= \frac{\overline{\beta}C\sum_i \frac{\chi_i\widehat{\eta}_i}{i\omega+A_i}+\sum_a \beta_a\widehat{\nu}_a}{i\omega+E+\overline{\beta}C\kappa\sum_i \frac{\chi_i^2 p_i}{A_i(i\omega+A_i)}}.
\end{aligned}
\tag{S4}
$$

As an illustration let us now compute the power spectrum of $\widehat{\mathcal{B}}$. To keep equations manageable, we define

$$
\begin{aligned}
f_i(\omega) &= \overline{\beta}C\frac{\chi_i}{(i\omega+A_i)},\\
g(\omega) &= (i\omega+E)+\overline{\beta}C\kappa\sum_i \frac{\chi_i^2 p_i}{A_i\,(i\omega+A_i)},
\end{aligned}
\tag{S5}
$$

and so we write the Fourier transform of $\tilde{\mathcal{B}}$ as

$$
\widehat{\mathcal{B}}(\omega) = \frac{\sum_i f_i\widehat{\eta}_i+\sum_a \beta_a\widehat{\nu}_a}{g},
\tag{S6}
$$

where f_i, β_a, $\widehat{\eta}_i$ and $\widehat{\nu}_a$ are all functions of ω. We then find

$$
\begin{aligned}
\mathcal{P}_{\mathcal{B}}(\omega) &= \left\langle \left(\frac{\sum_i f_i\widehat{\eta}_i+\sum_a \beta_a\widehat{\nu}_a}{g}\right)\left(\frac{\sum_i f_i^*\widehat{\eta}_i+\sum_a \beta_a\widehat{\nu}_a}{g^*}\right)\right\rangle\\
&= \frac{1}{|g|^2}\left(\sum_{i,j} f_i f_j^*\left\langle \widehat{\eta}_i\widehat{\eta}_j\right\rangle+\sum_{i,b} f_i\beta_b\left\langle \widehat{\eta}_i\widehat{\nu}_b\right\rangle+\sum_{a,j} f_j^*\beta_a\left\langle \widehat{\eta}_j\widehat{\nu}_a\right\rangle+\sum_{a,b}\beta_a\beta_b\left\langle \widehat{\nu}_a\widehat{\nu}_b\right\rangle\right)\\
&= \frac{1}{|g|^2}\left(\sum_i f_i f_i^*\left\langle \widehat{\eta}_i\widehat{\eta}_i\right\rangle+\sum_i\sum_{j\neq i} f_i f_j^*\left\langle \widehat{\eta}_i\widehat{\eta}_j\right\rangle+\sum_{i,b}(f_i+f_i^*)\,\beta_b\left\langle \widehat{\eta}_i\widehat{\nu}_b\right\rangle+\sum_a \beta_a^2\left\langle \widehat{\nu}_a\widehat{\nu}_a\right\rangle\right).
\end{aligned}
\tag{S7}
$$

The notation $*$ denotes complex conjugation. Substituting the noise correlators from Eqs. (S3),

$$
\begin{aligned}
\mathcal{P}_{\mathcal{B}}(\omega) &= \frac{1}{|g|^2}\Bigg(2\kappa\sum_i f_i f_i^* p_i-\kappa^2\sum_{i,j} f_i f_j^*\left(\frac{1}{A_i}+\frac{1}{A_j}\right)p_i p_j\\
&\qquad -\overline{\beta}\kappa C\sum_i (f_i+f_i^*)\left(\frac{\chi_i}{A_i}+\frac{\phi^\star}{D}\right)p_i+2\overline{\beta^2}C\phi^\star\Bigg),
\end{aligned}
\tag{S8}
$$

and, using Eq. (S6), we find

$$
\mathcal{P}_{\mathcal{B}}(\omega) = \frac{2\phi^\star C}{|g|^2}\left(\overline{\beta^2}-\frac{\overline{\beta}^2 C\kappa}{D}\sum_i \frac{\chi_i p_i A_i}{\omega^2+A_i^2}\right)-\frac{\left(\overline{\beta}C\kappa\right)^2}{|g|^2}\sum_{i,j}\frac{\chi_i p_i\chi_j p_j\,(A_i+A_j)\left(\omega^2+A_iA_j\right)}{A_iA_j(\omega^2+A_i^2)(\omega^2+A_j^2)},
\tag{S9}
$$

which is the PSD of $\mathcal{B}$, as also reported in Eq. (16) in the main text.

Following the same process, we can compute the PSD for the remaining quantities, $\mathfrak{X}$, I and S. We do not report all details, but only the final results

$$
\begin{aligned}
\mathcal{P}_{\mathfrak{X}}(\omega) &= \frac{1}{\overline{\beta}^2 C}\left[2\overline{\beta^2}\mathfrak{X}^\star + \left(\omega^2+E^2\right)\left(\frac{\mathcal{P}_{\mathcal{B}}}{C} - \frac{4\overline{\beta^2}\mathfrak{X}^\star}{|g|^2}\right)\right] \\
&+ \frac{2\kappa}{\overline{\beta}|g|^2}\sum_i \frac{\chi_i^2 p_i}{A_i\left(\omega^2+A_i^2\right)}\left[2\mathfrak{X}^\star\overline{\beta^2}\left(\omega^2 - EA_i\right) + \overline{\beta}A_i\left(\frac{A_i\mathfrak{X}^\star}{D\chi_i}+1\right)\left(\omega^2+E^2\right)\right] \\
&+ \frac{2\overline{\beta}C\kappa^2}{|g|^2}\sum_{i,j}\frac{\chi_i^2 p_i \chi_j^2 p_j\left[E\left(\omega^2+A_iA_j\right)+\omega^2\left(A_j-A_i\right)\right]}{A_iA_j\left(\omega^2+A_i^2\right)\left(\omega^2+A_j^2\right)}\left(\frac{A_i\mathfrak{X}^\star}{D\chi_i}+1\right),
\end{aligned}
$$

$$
\mathcal{P}_I(\omega) = \frac{\mathcal{P}_{\mathcal{B}}}{\overline{\beta}^2} + \frac{4C\mathfrak{X}^\star\left(\overline{\beta}^2 - \overline{\beta^2}\right)\left(DE+\omega^2\right)}{|g|^2\overline{\beta}^2\left(\omega^2+D^2\right)} + \frac{4\kappa C^2\mathfrak{X}^\star\left(\overline{\beta}^2-\overline{\beta^2}\right)}{\left(|g|^2\right)\left(\omega^2+D^2\right)\left(\overline{\beta}\right)}\sum_i\frac{\chi_i^2 p_i\left(DA_i-\omega^2\right)}{A_i\left(\omega^2+A_i^2\right)},
$$

$$
\begin{aligned}
\mathcal{P}_S(\omega) &= \frac{1}{\omega^2+\kappa^2}\left[\left(\frac{\omega^2+D^2}{\overline{\beta}^2}\right)\mathcal{P}_{\mathcal{B}} + 2\kappa\left(1-\kappa\sum_i\frac{p_i}{A_i}\right) - C\kappa\left(\frac{\mathfrak{X}^\star}{D}+\sum_i\frac{\chi_i p_i}{A_i}\right)\right] \\
&+ \frac{2C}{|g|^2\left(\omega^2+\kappa^2\right)}\left[-\frac{2\mathfrak{X}^\star\overline{\beta^2}}{\overline{\beta}^2}+\kappa\left(\frac{\mathfrak{X}^\star}{D}+\sum_i\frac{\chi_i p_i}{A_i}\right)\right]\left[DE+\omega^2-\frac{|g|^2}{2}+\overline{\beta}C\kappa\sum_j\frac{\chi_j^2 p_j\left(DA_j-\omega^2\right)}{A_j\left(\omega^2+A_j^2\right)}\right] \\
&+ \frac{2C\kappa}{|g|^2\left(\omega^2+\kappa^2\right)}\sum_i\frac{\chi_i p_i}{A_i\left(\omega^2+A_i^2\right)}\left[C\left(\frac{A_i\mathfrak{X}^\star}{D}+\chi_i\right)-2A_i+\kappa\left(1+A_i\sum_k\frac{p_k}{A_k}\right)\right] \\
&\qquad\left\{A_iDE+\omega^2\left(A_i+E-D\right)+\overline{\beta}C\kappa\sum_j\frac{\chi_j^2 p_j\left[D\left(A_iA_j+\omega^2\right)+\omega^2\left(A_j-A_i\right)\right]}{A_j\left(\omega^2+A_j^2\right)}\right\}. \quad \text{(S10)}
\end{aligned}
$$

The power spectra of fluctuations for the individual subgroups of infectives and susceptibles are found as

$$
\begin{aligned}
\mathcal{P}_{x_i}(\omega) &= \frac{1}{\omega^2+A_i^2}\left[\left(\frac{\kappa\chi_i p_i}{A_i}\right)^2\mathcal{P}_{\mathcal{B}} + 2\kappa\left(1-\frac{\kappa p_i}{A_i}\right)p_i\right] \\
&+ \frac{2\overline{\beta}C\kappa^2\chi_i p_i^2}{|g|^2A_i\left(\omega^2+A_i^2\right)}\left[\frac{\mathfrak{X}^\star}{D}+\frac{\chi_i\left(\omega^2-A_i^2\right)}{A_i\left(\omega^2+A_i^2\right)}+\frac{\kappa}{A_i}\sum_j\frac{\chi_j p_j\left(A_i+A_j\right)}{\omega^2+A_j^2}\right]\left(E+\sum_k\frac{\overline{\beta}C\kappa\chi_k^2 p_k}{\omega^2+A_k^2}\right) \\
&+ \frac{2\overline{\beta}C\kappa^2\chi_i p_i^2\omega^2}{|g|^2A_i\left(\omega^2+A_i^2\right)}\left[\frac{2\chi_i}{\omega^2+A_i^2}-\frac{\kappa}{A_i}\sum_j\frac{\chi_j p_j\left(A_i+A_j\right)}{A_j\left(\omega^2+A_j^2\right)}\right]\left(1-\sum_k\frac{\overline{\beta}C\kappa\chi_k^2 p_k}{A_k\left(\omega^2+A_k^2\right)}\right), \quad \text{(S11)}
\end{aligned}
$$

$$
\mathcal{P}_{y_a}(\omega) = q_a^2\mathcal{P}_I + \frac{2C\mathfrak{X}^\star q_a\left(1-q_a\right)}{\omega^2+D^2}. \quad \text{(S12)}
$$

S3 Phase Lag

In order to explore the the phase lag we use the so-called complex coherence function, $\mathcal{CCF}_{ij}$, between subgroups i and j, defined as

$$\mathcal{CCF}_{ij}(\omega) = \frac{\left\langle \widehat{x}_i \widehat{x}_j^* \right\rangle}{\sqrt{\left\langle \widehat{x}_i \widehat{x}_i^* \right\rangle \left\langle \widehat{x}_j \widehat{x}_j^* \right\rangle}} = \frac{\mathcal{P}_{x_i x_j}}{\sqrt{\mathcal{P}_{x_i} \mathcal{P}_{x_j}}}, \tag{S13}$$

where $\widehat{x}_i$ and $\mathcal{P}$ are functions of ω.

For $i \neq j$ this is in general a complex-valued function (of ω). The argument of $\mathcal{CCF}_{ij}$, given by

$$\mathfrak{L}_{x_i x_j}(\omega) = \tan^{-1} \frac{\mathrm{Im}\ \mathcal{CCF}_{ij}(\omega)}{\mathrm{Re}\ \mathcal{CCF}_{ij}(\omega)} = \tan^{-1} \frac{\mathrm{Im}\ \mathcal{P}_{x_i x_j}(\omega)}{\mathrm{Re}\ \mathcal{P}_{x_i x_j}(\omega)}, \tag{S14}$$

is known as the phase spectrum; it describes the phase-lag between the time series $x_i(t)$ and $x_j(t)$[2].

The cross spectra of the population in the susceptible classes normalized with respect to the total population ($x_i = n_i/N$) is given by

$$\mathcal{P}_{x_i x_j}(\omega) = \left\langle \widehat{x}_i \widehat{x}_j^* \right\rangle = \left\langle \left(\frac{-\chi_i x_i^\star \widehat{\mathcal{B}} + \widehat{\eta}_i}{i\omega + A_i} \right) \left(\frac{-\chi_j x_j^\star \widehat{\mathcal{B}}^* + \widehat{\eta}_j}{-i\omega + A_j} \right) \right\rangle. \tag{S15}$$

This can be written as

$$\mathcal{P}_{x_i x_j}(\omega) = \frac{\left(\omega^2 + A_i A_j\right) \mathcal{W}_{ij} - \omega \left(A_i - A_j\right) \mathcal{U}_{ij}}{\left(\omega^2 + A_i^2\right)\left(\omega^2 + A_j^2\right)} + i\ \frac{\left(\omega^2 + A_i A_j\right) \mathcal{U}_{ij} + \omega \left(A_i - A_j\right) \mathcal{W}_{ij}}{\left(\omega^2 + A_i^2\right)\left(\omega^2 + A_j^2\right)}, \tag{S16}$$

where we introduced the notation

$$\begin{aligned} \mathcal{U}_{ij}(\omega) &= \chi_j x_j^\star \mathrm{Im} \left\langle \widehat{\eta}_i \widehat{\mathcal{B}} \right\rangle - \chi_i x_i^\star \mathrm{Im} \left\langle \widehat{\eta}_j \widehat{\mathcal{B}} \right\rangle, \\ \mathcal{W}_{ij}(\omega) &= \left(\chi_i \chi_j x_i^\star x_j^\star\right) \mathcal{P}_{\mathcal{B}} + \left\langle \widehat{\eta}_i \widehat{\eta}_j \right\rangle - \chi_j x_j^\star \mathrm{Re} \left\langle \widehat{\eta}_i \widehat{\mathcal{B}} \right\rangle - \chi_i x_i^\star \mathrm{Re} \left\langle \widehat{\eta}_j \widehat{\mathcal{B}} \right\rangle. \end{aligned} \tag{S17}$$

From these we obtain the phase lag as

$$\mathfrak{L}_{x_i x_j}(\omega) = \tan^{-1} \frac{\omega \left(A_i - A_j\right) \mathcal{W}_{ij} + \left(\omega^2 + A_i A_j\right) \mathcal{U}_{ij}}{\left(\omega^2 + A_i A_j\right) \mathcal{W}_{ij} - \omega \left(A_i - A_j\right) \mathcal{U}_{ij}}, \tag{S18}$$

which yields the theoretical lines in Fig. 9a.

To explore the phase lag between the susceptible subgroups when normalized by the total susceptible population ($x'_i = n_i/NS$), we first need to compute the cross-spectra of the renormalized signals $\mathcal{P}_{x'_i x'_j}(\omega)$. As in Section 3.1, we start from the ansatz

$$\frac{n_i}{NS} = x'_i + \frac{1}{\sqrt{N}} \tilde{x}'_i. \tag{S19}$$

We then have

$$\frac{n_i}{NS} = \frac{n_i/N}{S} = \frac{x_i + \frac{1}{\sqrt{N}} \tilde{x}_i}{S + \frac{1}{\sqrt{N}} \tilde{S}} \equiv x'_i + \frac{1}{\sqrt{N}} \tilde{x}'_i, \tag{S20}$$

and so (after expanding in $1/\sqrt{N}$)

$$\tilde{x}'_i = \frac{S^\star \tilde{x}_i - x_i^\star \tilde{S}}{\left(S^\star\right)^2}. \tag{S21}$$

In Fourier space this turns into

$$\widehat{x}_i' = \frac{S^\star \widehat{x}_i - x_i^\star \widehat{S}}{\left(S^\star\right)^2}. \tag{S22}$$

For the cross spectra we then find

$$\mathcal{P}_{x_i' x_j'}(\omega) = \langle \widehat{x}_i' \widehat{x}_j'^* \rangle = \left\langle \frac{\left(S^\star \widehat{x}_i - x_i^\star \widehat{S}\right)\left(S^\star \widehat{x}_j^* - x_j^\star \widehat{S}^*\right)}{\left(S^\star\right)^4} \right\rangle, \tag{S23}$$

which can be rewritten as

$$\mathcal{P}_{x_i' x_j'}(\omega) = \frac{1}{\left(S^\star\right)^3}\left(S^\star \mathrm{Re}\left[\mathcal{P}_{x_i x_j}\right] + \frac{\kappa^2 p_i p_j}{S^\star A_i A_j}\mathcal{P}_S - \frac{Y_{ij\,R} + Y_{ji\,R}}{\overline{\beta}\left(\omega^2+\kappa^2\right)}\right) + \frac{i}{\left(S^\star\right)^3}\left(S^\star \mathrm{Im}\left[\mathcal{P}_{x_i x_j}\right] - \frac{Y_{ij\,I} - Y_{ji\,I}}{\overline{\beta}\left(\omega^2+\kappa^2\right)}\right). \tag{S24}$$

We have introduced the notation $Y_{ij\,R} = \mathrm{Re}[Y_{ij}]$ and $Y_{ij\,I} = \mathrm{Im}[Y_{ij}]$ with

$$\begin{aligned} Y_{ij}(\omega) \quad = \quad & \frac{\left(\omega^2 + i\omega\left(\kappa - A_j\right) + \kappa A_j\right)\kappa p_i}{\left(\omega^2 + A_j^2\right)A_i}\left\{\frac{\kappa \chi_j p_j}{A_j}\left(D\mathcal{P}_{\mathcal{B}} - \sum_a \beta_a \mathrm{Re}\left\langle \widehat{\nu}_a \widehat{\mathcal{B}}\right\rangle - \overline{\beta}\sum_k \mathrm{Re}\left\langle \widehat{\eta_k \mathcal{B}}\right\rangle\right)\right. \\ & \left. \omega \mathrm{Im}\left\langle \widehat{\eta}_j \widehat{\mathcal{B}}\right\rangle - D\mathrm{Re}\left\langle \widehat{\eta}_j \widehat{\mathcal{B}}\right\rangle - \kappa\overline{\beta}p_j\left[C\left(\frac{\chi_j}{A_j} + \frac{\mathfrak{X}^\star}{D}\right) - 2 + \frac{\kappa}{A_j} + \kappa\sum_k\left(\frac{p_k}{A_k}\right)\right]\right\} \\ & + i\frac{\left[\omega^2 + i\omega\left(\kappa - A_j\right) + \kappa A_j\right]\kappa p_i}{\left(\omega^2 + A_j^2\right)A_i}\left[\frac{\kappa\chi_j p_j}{A_j}\left(\omega\mathcal{P}_{\mathcal{B}} + \sum_a \beta_a \mathrm{Im}\left\langle \widehat{\nu}_a \widehat{\mathcal{B}}\right\rangle + \overline{\beta}\sum_k \mathrm{Im}\left\langle \widehat{\eta_k \mathcal{B}}\right\rangle\right)\right. \\ & \left. -\omega\mathrm{Re}\left\langle \widehat{\eta}_j \widehat{\mathcal{B}}\right\rangle - D\,\mathrm{Im}\left\langle \widehat{\eta}_j \widehat{\mathcal{B}}\right\rangle\right]. \end{aligned} \tag{S25}$$

From these, we can find the phase-lag as

$$\mathfrak{L}_{x_i' x_j'}(\omega) = \tan^{-1}\frac{S^\star \mathrm{Im}\left[\mathcal{P}_{x_i x_j}\right] - \frac{Y_{ij\,I} - Y_{ji\,I}}{\overline{\beta}\left(\omega^2+\kappa^2\right)}}{S^\star \mathrm{Re}\left[\mathcal{P}_{x_i x_j}\right] + \frac{\kappa^2 p_i p_j}{S^\star A_i A_j}\mathcal{P}_S - \frac{Y_{ij\,R} + Y_{ji\,R}}{\overline{\beta}\left(\omega^2+\kappa^2\right)}}. \tag{S26}$$

This expression was used to obtain the analytical predictions shown in Fig. 9b.

S4 Table of Symbols

		Meaning	Defined in Eqs.
Symbols	N	Total population size.	
	K	Number of susceptible subgroups.	
	M	Number of infective subgroups.	
	S_i	Susceptible subgroup i.	
	I_a	Infective subgroup a.	
	n_i	Number of individuals of type S_i.	
	m_a	Number of individuals in class I_a.	
	p_i	Probability of being assigned a susceptibility χ_i at birth.	
	q_a	Probability of being assigned an infectiousness β_a upon infection.	
	χ_i	Susceptibility of subgroup i.	
	β_a	Infectiousness of subgroup a.	
	ρ	Recovery rate.	
	κ	Death/birth rate.	
	ξ	Spontaneous infection rate.	
	$\overline{\chi}$	Mean susceptibility at birth.	(2)
	$\mathfrak{X}$	Aggregate susceptibility of the population.	(2)
	$\overline{\beta}$	Mean infectiousness upon infection.	(3)
	$\mathcal{B}$	Total 'infective power' in the population.	(3)
	x_i	Fraction of susceptible individuals in subgroup i in the limit of infinite system size.	
	y_a	Fraction of infected individuals in subgroup a in the limit of infinite system size.	
	S	Total density of susceptible individuals in the population.	(5)
	I	Total density of infective individuals in the population.	(5)
	$\mathfrak{X}_n$	Nth moment of the aggregate susceptibility.	
	$\overline{\chi^n}$	Nth moment of susceptibilities at birth.	
	A_i, C, D, E	Notation introduced to simplify equations.	(15)
	$\mathcal{P}_z$	Power spectral density of z.	(16)
	g	Notation introduced to simplify equations.	(17)
	ω_d	Dominant cycle frequency.	(18)
	$\overline{\beta^2}$	Second moment of the infectiousness assigned upon infection.	(19)
	$\mathbb{S}$	Sharpness of the PSD.	(20)
	$\mathcal{L}_{z_1 z_2}$	Phase lag between signals z_1 and z_2.	(21)
Accents	$\overline{\chi}$	Expected value at birth or upon infection.	(2), (3)
	$\dot{x}$	Deterministic evolution (time derivative).	(4), (6), (7)
	$x^\star$	Deterministic fixed point.	(9), (10)
	$\tilde{x}$	Stochastic fluctuations about the deterministic fixed point.	(11), (12)
	$\widehat{x}$	Fourier transform with respect to time.	(13), (14)
	x^*	Complex conjugate.	

References

1. van Kampen, N. G. *Stochastic processes in physics and chemistry* (Elsevier, Amsterdam, 1992), 3rd edn.
2. Rozhnova, G., Nunes, A. & McKane, A. J. Phase lag in epidemics on a network of cities. *Phys. Rev. E* **85** (2012). DOI 10.1103/PhysRevE.85.051912.
